# Changes in frailty among community-dwelling Chinese older adults and its predictors: evidence from a two-year longitudinal study

**DOI:** 10.1186/s12877-020-01530-x

**Published:** 2020-04-10

**Authors:** Bo Ye, Hao Chen, Limei Huang, Ye Ruan, Shige Qi, Yanfei Guo, Zhezhou Huang, Shuangyuan Sun, Xiuqin Chen, Yan Shi, Junling Gao, Yonggen Jiang

**Affiliations:** 1 School of Public Health, Fudan University, PO Box 248, 138 Yixueyuan Road, Shanghai, 200032 China; 2 Songjiang Center of Disease Prevention and Control, Shanghai, 201620 China; 3 Shanghai Municipal Center for Disease Control and Prevention, Shanghai, 200336 China; 4 The National Center for Chronic and Noncommunicable Disease Control and Prevention, Beijing, 100050 China

**Keywords:** Frailty, Frailty index, Change of frailty, Transitions, Lifestyle, Predictors, Older adults, Community-based

## Abstract

**Background:**

It is important to clarify the transitions and related factors of frailty for prevention of frailty. We evaluated the transitions of frailty among community-dwelling older adults and examined the predictors of the transitions.

**Methods:**

A cohort study was conducted among 3988 community residents aged ≥60 years during 2015 and 2017. A multiple deficits approach was used to construct the Frailty Index (FI) according to the methodology of FI construction, and sociodemographic characteristics and lifestyles were also collected in 2015. After 2-year follow-up, the transitions of frailty between baseline and were evaluated. Multinomial logistic regressions were used to examine associations between predictors and the transitions of frailty.

**Results:**

The proportion of robust, prefrail, and frail was 79.5, 16.4, and 4.1% among 3988 participants at baseline, which changed to 68.2, 23.0, and 8.8% after 2 years with 127 deaths and 23 dropped out. Twelve kinds of transitions from the three frailty statuses at baseline to four outcomes at follow-up (including death) significantly differed within each of gender and age group, as well between genders and age groups. Among these, 7.8% of prefrail or frail elders improved, 70.0% retained their frailty status, and 22.2% of robust or prefrail elders worsened in frailty status. In multivariable models, age was significantly associated with changes in frailty except for in the frail group; higher educational level and working predicted a lower risk of robust worsening. Of the lifestyle predictors, no shower facilities at home predicted a higher risk of robust worsening; more frequent physical exercise predicted a lower risk of robust worsening and a higher chance of frailty improvement; more frequent neighbor interaction predicted a lower risk of robust worsening and prefrail worsening; and more frequent social participation predicted a higher chance of prefrail improvement.

**Conclusions:**

The status of frailty was reversible among community-dwelling elderly, and sociodemographic and lifestyle factors were related to changes in frailty. These findings help health practitioners to recognize susceptible individuals in a community and provide health promotional planning to target aged populations.

## Background

Frailty is an unstable status with the age-related loss of physiological reserves and disorders in homeostatic systems [1, 2]. The presence of frailty is not only symptomatic in older individuals, but it also renders them more prone to downstream changes in long-term health outcomes, such as disability, hospitalization, institutionalization, and mortality [1, 3–6]. In the absence of a gold standard, the two approaches most widely used are frailty phenotype (FP) [1] and frailty index (FI) [7]. FP is defined on the basis of weight loss, exhaustion, physical activity, walk time, and grip strength, while FI is defined as an individual’s accumulated proportion of listed health-related deficits. Based on the used definitions, the prevalence of frailty ranges from 4.0 to 59.1% in community-dwelling older adults, [8] and both FP and FI can effectively predict adverse outcomes [9]. An exponential correlation has been shown between FI and age, [7, 10] and the heterogeneity of physiological reserves trends to be greater in later life [11]. The approach of FI represents a continuous status of health, [12] which is more likely to demonstrate the dynamic nature of frailty in the general aged population. Frailty among older adults is generally agreed to be a dynamic status [13–15] that is inevitable with increasing age but reversible, [16, 17] and it may represent an intermediate stage between healthiness and the end of life as a biological age [1, 10]. It is conceivable that FI could also represent the cumulative effect of multiple individuals and environmental factors from a health ecological perspective. Understanding the characteristics related to frailty transitions will allow for better future health practice and healthcare strategies.

Several studies have reported on the natural transitions in frailty status and their factors, which are mostly associations with sociodemographic factors and health status [17–24]. For instance, those who are older, [17, 19, 23, 24] have fewer years of education, [16] have diabetes and previous stroke, [17] have poor functional performance, [19, 21, 24] and have cognitive impairment [22] are associated with frailty worsening. These findings are helpful for health practitioners to recognize susceptible individuals, though they seem inadequate to provide health promotional planning for community aged populations. Identifying the social and behavioral factors that may worsen or improve frailty would contribute to establishing appropriate measures to prevent or delay frailty progression in a broader-aged population. Numerous studies have shown an association between lifestyle factors and frailty [25–28]. However, few studies have examined the relationship between potential social and behavioral factors and changes in frailty.

Recently, a longitudinal study provided evidence that older Chinese living in communities that have a higher percentage of green space had a higher likelihood of improvement in frailty status, and physical activity presented a mediation effect [29]. It is widely recognized that there are close associations between social and behavioral factors and health outcomes, and various studies have demonstrated them to be effective interventions for the frail elderly, including physical exercise and social support [2, 30–32]. It is reasonable to believe that an individual’s lifestyle has an important influence on changes in frailty. Moreover, a 15-year longitudinal study showed that an index constructed by multiple lifestyle protective factors is associated with a lower risk of worsening frailty and a greater chance of recovery among Chinese community-dwelling older adults [33]. However, the independent effects of each factor are unknown, and additional lifestyle factors that are easy to modify need to be explored and their protective effects on frailty tested in future studies.

Based on these considerations, we investigated how changes in frailty occurred among community-dwelling older Chinese through a 2-year cohort study, and lifestyle factors associated with changes in frailty were identified.

## Methods

### Study design and participants

This was a longitudinal observational study among Chinese older adults living in the community of Shanghai. Multi-stage random sampling was used to select subjects. Two of 11 streets were randomly selected, and then four communities were randomly selected from each street. Simple random sampling was used to select family, in which older adults aged ≥60 years lived in through a household registration information system. Residents aged ≥60 years among selected families were all approached to participate in the investigation. Written informed consent was obtained from each study participant, and the Research Ethics Committee of the Division for the Prevention and Control of Chronic Non-communicable Diseases, China Center for Disease Control and Prevention, approved the study protocol. A total of 4050 participants aged ≥60 years were originally recruited for an investigation of chronic diseases and geriatric syndromes in 2015. After eliminating invalid answers to questionnaires, the valid response proportion was 98.5% (3988/4050). Finally, the average age and its standard deviation (SD) of participants was 69.38 (7.06), with 43.5% male, 79.8% married, and more than half never educated (as shown in Table 1). During the next 2 years, there were 127 deaths and 23 participants lost to follow-up. Of those who were lots to follow-up, were unable to recontact them and some may have emigrated to another city. Five of these participants had prefrail and 18 had robust status.

**Table 1:** Description of predictors according to frailty status at baseline

Predictors	Total	Robust (≤ 0.10)	Prefrail (≤ 0.21)	Frail (> 0.21)	*P* value
	*n* = 3988, n (%)	*n* = 3169, n (%)	*n* = 656, n (%)	*n* = 163, n (%)	
**Gender (Male)**		1500 (47.3)	187 (28.5)	49 (30.1)	< 0.001
**Age, year (Mean ± SD)**	69.38 ± 7.06	68.17 ± 6.37	73.23 ± 7.17	77.47 ± 8.38	< 0.001 ^a^
**Age group, year**					< 0.001
60–69	2314 (58.0)	2056 (65.1)	216 (32.9)	33 (20.2)	
70–79	1237 (31.0)	883 (27.9)	297 (45.3)	57 (35.0)	
80+	437 (11.0)	221 (7.0)	143 (21.8)	73 (44.8)	
**Marital status (Married)**	3181 (79.8)	2645 (83.5)	451 (68.8)	85 (52.1)	< 0.001
**Educational level**					< 0.001
Illiteracy	2251 (56.4)	1592 (50.3)	519 (79.1)	140 (85.9)	
Primary school	1176 (29.5)	1069 (33.7)	93 (14.2)	14 (8.6)	
Junior high school or above	561 (14.1)	508 (16.0)	44 (6.7)	9 (5.5)	
**Working (Yes)**	629 (15.8)	567 (17.9)	58 (8.8)	4 (2.5)	< 0.001
**Live alone (Yes)**	325 (8.1)	218 (6.9)	80 (12.2)	27 (16.6)	< 0.001
**Shower facility at home (No)**	381 (9.6)	248 (7.8)	108 (16.5)	25 (15.3)	< 0.001
**Annual physical examination (No)**	357 (9.0)	249 (7.9)	62 (9.5)	46 (28.2)	< 0.001
**Cigarette smoking**					< 0.001
Nonsmoker	2662 (66.8)	2018 (63.6)	514 (78.4)	130 (79.8)	
Past smoker	488 (12.2)	392 (12.4)	74 (11.3)	22 (13.5)	
Current smoker	838 (21.0)	759 (24.0)	68 (10.4)	11 (6.7)	
**Alcohol intake (Yes)**	749 (18.8)	686 (21.6)	60 (9.1)	3 (1.8)	< 0.001
**Daily tea (Yes)**	1370 (34.4)	1198 (37.8)	149 (22.7)	23 (14.1)	< 0.001
**Reading (Yes)**	595 (14.9)	542 (17.1)	49 (7.5)	4 (2.5)	< 0.001
**Playing cards or mahjong (Yes)**	917 (23.0)	813 (25.7)	97 (14.8)	7 (4.3)	< 0.001
**Physical exercise**					< 0.001
Almost never	992 (24.9)	747 (23.6)	166 (25.3)	79 (48.5)	
Several times per week	805 (20.2)	606 (19.1)	166 (25.3)	33 (20.2)	
Everyday	2191 (54.9)	1816 (57.3)	324 (49.4)	51 (31.3)	
**Meeting with children**					0.073
Almost never	801 (20.0)	636 (20.1)	143 (21.8)	22 (13.5)	
Several times per week	1757 (44.1)	1411 (44.5)	277 (42.2)	69 (42.3)	
Everyday	1430 (35.9)	1122 (35.4)	236 (36.0)	72 (44.2)	
**Neighbor interaction**					< 0.001
Almost never	517 (13.0)	384 (12.1)	96 (14.6)	37 (22.7)	
Several times per week	505 (12.7)	376 (11.9)	103 (15.7)	256 (16.0)	
Everyday	2966 (74.3)	2409 (76.0)	457 (69.7)	100 (61.3)	
**Social participation**					< 0.001
Almost never	2915 (73.1)	2308 (72.8)	474 (72.3)	133 (81.6)	
Several times per month	522 (13.1)	392 (12.4)	107 (16.3)	23 (14.1)	
Several times per week	551 (13.8)	469 (14.8)	75 (11.4)	7 (4.3)	

^a^*P* value for ANOVA

### Frailty index construction and frailty transitions

According to the methodology of FI construction, a multiple deficits approach was used to construct the FI. Deficits were defined as “symptoms, signs, disabilities, and diseases,” which prevalence must increase with age [34]. Thirty-six eligible items were eventually selected covering the self-reported presence of current diseases (5 items), cognitive and mental symptoms (9 items), ability in the activities of daily living (15 items), as well as physical and neurological signs (7 items) (Details in Additional file 1). All items for FI were dichotomized into the presence (1) or absence (0) of a frailty deficit. The FI was calculated as the proportion of the number of deficits for an individual to the maximum total number of deficits. According to previous studies, [23, 35] we categorized the FI score into a three-level variable: robust (FI ≤ 0.10), prefrail (0.10 < FI ≤ 0.21), and frail (FI >  0.21). Frailty transitions were included for the three kinds of frailty status (robust, prefrail, and frail) changing into to each other among survivors after 2 years, which were defined as worsening, stability, or improvement. Outcomes also included death from each of the three kinds of frailty status.

### Predictors

Sociodemographic factors included age (Years), gender (Male/Female), educational level (Illiteracy/Primary school/Junior high school or above), marital status (Married/Unmarried; ‘Unmarried’ included never married, divorced, and widowed), and working status (Yes/No; ‘Yes’ means an individual still works for payment or for free, such as a volunteer, and ‘No’ means an individual has retired or has no work).

Lifestyle factors included living alone (Yes/No), has a shower facility at home (Yes/No), annual physical examination (Yes/No), cigarette smoking (Current smoker, Former smoker, Nonsmoker), alcohol intake (Yes/No; ‘Yes’ means an individual drinks sometimes or more often, and ‘No’ means an individual never drinks), daily tea (Yes/No), reading (Yes/No; ‘Yes’ means an individual reads books or newspapers sometimes or more often, and ‘No’ means an individual almost never reads), plays cards or mahjong (Yes/No; ‘Yes’ means an individual plays cards or mahjong sometimes or more often, and ‘No’ means an individual almost never plays), physical exercise (Almost never/Several times per week/Everyday; ‘Almost never’ means an individual does exercises over 10 min for only several times per month or less, ‘Several times per week’ means an individual does exercises over 10 min for 1–6 days, ‘Everyday’ means an individual does exercises over 10 min everyday), meeting with children (Almost never/Several times per week/Everyday; ‘Almost never’ means an individual meets with children for only several times per month or less), neighbor interaction (Almost never/Several times per week/Everyday; ‘Almost never’ means an individual interacts with neighbors over 10 min for only several times per month or less), and social participation (Almost never/Several times per month/Several times per week; ‘Almost never’ means an individual takes part in various social activities for several times per year or never). Alcohol intake was selected single frequency domain from quantity frequency scale (QF), regularly screening patients for alcohol problems by primary care doctors, and was degreed answer into non-drinker (never drinking at all) and drinker (some times and more often) [36]. Physical exercise was designed categorized answers according to absolute physical measurements, which aims to quickly classify physical exercise level [37]. The number and proportion of each lifestyle variable is presented in Table 1.

### Statistical analysis

Descriptive statistics for demographic and lifestyle variables are presented as the frequency and percentage, continuous variables are described as means and SD, and the Chi-square test was used to evaluate the distribution of the three kinds of frailty status at baseline between groups according to demographics and lifestyle. The health outcomes after 2 years and the three kinds of frailty transitions were also described and compared according to gender and age. Multinomial logistic regressions were used to explain whether the 2-year change of frailty was associated with lifestyle factors, with unadjusted and adjusted coefficients both reported. Windows-based SPSS version 22.0 (SPSS Inc., Chicago, IL, USA) was used for all of the statistical analysis, and a *P* value of less than 0.05 was considered to be statistically significant.

## Results

Table 1 showed the participants’ sociodemographic and lifestyle factors, and a comparison of these factors among the three frailty statuses at baseline. Prefrail and frail female elders were more common than males, while there were similar proportions of robust male and female elders. The average age of the frail, prefrail, and robust elders significantly increased. Except for meeting with children, all of the lifestyle factors were correlated with the frailty status at baseline. As expected, lifestyle protective factors included married, higher educational level, working, daily tea, reading, playing cards or mahjong, physical exercise, neighbor interaction, and social participation; and risk factors were live alone, lacking shower facilities at home, and no annual physical examination. However, there were higher proportions of current smokers and alcohol drinkers among the robust elders.

**Fig. 1:**
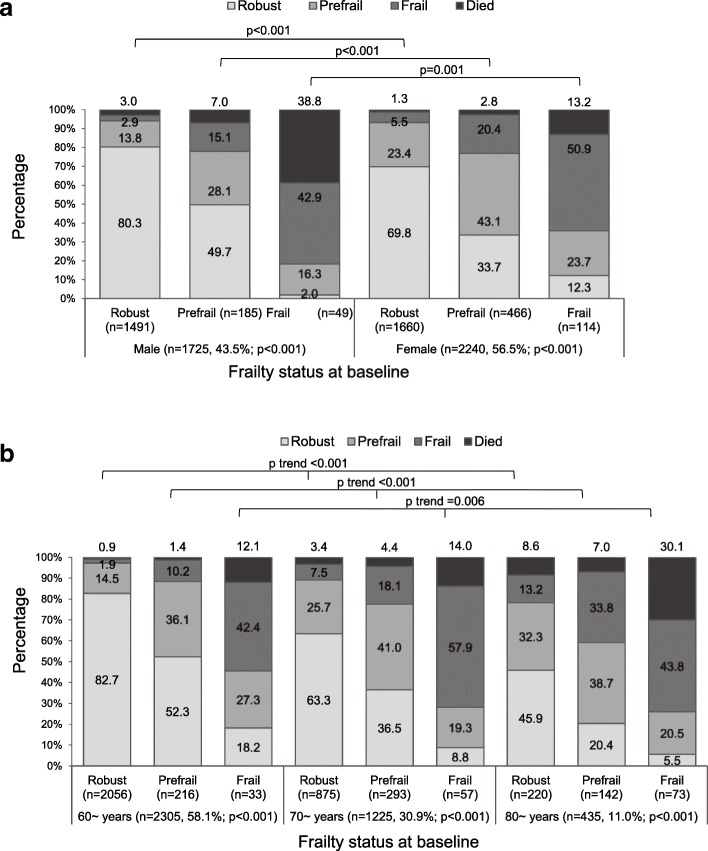
**a** Transitions of frailty status from baseline to follow-up according to gender. **b** Transitions of frailty status from baseline to follow-up according to age

There were twelve kinds of transitions from baseline of the three frailty statuses to 2-year follow-up of four outcomes (including 127 deaths). Figure 1 shows the results of frailty status transitions according to gender (a) and age (b). The frailty status transitions significantly differed within each of gender and age group and also significantly differed between gender and age groups. Table 2 shows the changes in frailty among the 2-year survivors: 7.8% (299/3838), 70.0% (2687/3838), and 22.2% (852/3838) of elders improved (improvement), stayed the same (stability), and worsened (worsening) in frailty status, respectively. Among the baseline robust and prefrail elders, female elders (robust: 29.3% vs. 17.2%; prefrail: 21.0% vs. 16.3%), and older elders (robust: 49.8% vs. 34.4% vs. 16.6%) had a higher risk of frailty worsening, while there were no statistically significant differences between gender and age groups among the baseline frail elders. In further multivariate analyses, there were no significant associations between changes in frailty and gender, and the associations between age and the three changes in frailty were significant except for in the frail group.

**Table 2:** Transitions in frailty status among the survivors according to gender and age

Characteristics	Frailty transitions during 2 years, n (%)						
	Robust (*n* = 3084, 80.4%)		Prefrail (*n* = 625, 16.3%)			Frail (*n* = 129, 3.4%)	
	Stability	Worsening	Improvement	Stability	Worsening	Improvement	Stability
**Gender**							
Male	1197 (82.8)	249 (17.2)	92 (53.5)	52 (30.2)	28 (16.3)	9 (30.0)	21 (70.0)
Female	1158 (70.7)	480 (29.3)	157 (34.7)	201 (44.4)	95 (21.0)	41 (41.4)	58 (58.6)
*P* value	< 0.001		< 0.001			0.261	
**Age, years**							
60~	1700 (83.4)	338 (16.6)	113 (53.1)	78 (36.6)	22 (10.3)	15 (51.7)	14 (48.3)
70~	554 (65.6)	291 (34.4)	107 (38.2)	120 (42.9)	53 (18.9)	16 (32.7)	33 (67.3)
80~	101 (50.2)	100 (49.8)	29 (22.0)	55 (41.7)	48 (36.4)	19 (37.3)	32 (62.7)
*P* value	< 0.001		< 0.001			0.238	

Table 3 shows the results of univariate (unadjusted models) and multivariate (adjusted models) analyses between lifestyle predictors and changes in frailty. Unadjusted and adjusted ORs and their 95% CI were used to estimate the risk of worsening and improvement compared to stability among the three kinds of baseline frailty status, respectively. In the unadjusted models, except for annual physical examination and meeting with children, all of the lifestyle factors were associated with robust worsening, while only neighbor interaction was associated with prefrail worsening. In addition, marital status, education, living alone, shower facilities at home, smoking, daily tea, reading, and social participation were associated with prefrail improvement, and physical exercise and neighbor interaction were associated with frail improvement.

**Table 3:** Multiple logistic regression for changes in frailty

Predictors	Robust worsening, OR (95%CI)		Prefrail worsening, OR (95%CI)		Prefrail improvement, OR (95%CI)		Frail improvement, OR (95%CI)	
	Unadjusted model ^a^	Adjusted model ^b^	Unadjusted model ^a^	Adjusted model ^b^	Unadjusted model ^a^	Adjusted model ^b^	Unadjusted model ^a^	Adjusted model ^b^
**Gender** (Female)	1.99^***^ (1.68, 2.37)	1.30 (0.95, 1.79)	0.88 (0.52, 1.48)	0.56 (0.21, 1.52)	0.44^***^ (0.30, 0.66)	0.65 (0.28, 1.49)	1.65 (0.69, 3.97)	0.84 (0.19, 3.68)
**Age** (vs 60~ years)								
70~ years	2.64^***^ (2.20, 3.18)	2.10^***^ (1.72, 2.58)	1.57 (0.88, 2.78)	1.81 (0.98, 3.34)	0.62^*^ (0.42, 0.91)	0.70 (0.45, 1.07)	0.45 (0.18, 1.16)	0.36 (0.12, 1.14)
80~ years	4.98^***^ (3.69, 6.72)	3.30^***^ (2.36, 4.60)	3.09^***^ (1.68, 5.70)	3.89^***^ (1.87, 8.09	0.36^***^ (0.21, 0.62)	0.40^**^ (0.21, 0.75)	0.55 (0.22, 1.40)	0.53 (0.15, 1.84)
**Marital status** (Married)	0.47^***^ (0.38, 0.58)	0.83 (0.63, 1.09)	1.03 (0.65, 1.61)	1.29 (0.66, 2.53)	1.66^**^ (1.13, 2.45)	0.84 (0.47, 1.50)	0.77 (0.38, 1.57)	1.10 (0.35, 3.47)
**Educational level** (vs Illiteracy)								
Primary school	0.46^***^ (0.38, 0.56)	0.63^***^ (0.51, 0.78)	0.88 (0.43, 1.80)	0.98 (0.43, 2.23)	2.11^**^ (1.27, 3.50)	1.51 (0.86, 2.67)	0.76 (0.18, 3.18)	0.78 (0.10, 6.01)
Junior high school or above	0.32^***^ (0.24, 0.42)	0.46^***^ (0.33, 0.64)	1.23 (0.44, 3.47)	1.04 (0.27, 4.04)	3.19^**^ (1.50, 6.80)	2.02 (0.80, 5.12)	0.60 (0.11, 3.25)	0.66 (0.07, 6.08)
**Working** (Yes)	0.42^***^ (0.32, 0.55)	0.60^***^ (0.45, 0.80)	0.47 (0.17, 1.27)	0.56 (0.20, 1.61)	1.51 (0.84, 2.72)	1.33 (0.71, 2.51)	1.60 (0.22, 11.77)	2.00 (0.15, 27.26)
**Live alone** (Yes)	1.73^***^ (1.28, 2.34)	0.94 (0.63, 1.41)	0.65 (0.33, 1.26)	0.55 (0.22, 1.41)	0.53^*^ (0.30, 0.93)	0.58 (0.26, 1.30)	1.13 (0.58, 3.50)	1.69 (0.39, 7.40)
**Shower facility at home** (No)	1.91^***^ (1.45, 2.53)	1.39^*^ (1.02, 1.90)	0.88 (0.50, 1.55)	0.89 (0.47, 1.70)	0.61^*^ (0.37, 0.99)	0.71 (0.41, 1.24)	0.94 (0.32, 2.77)	1.42 (0.34, 5.87)
**Annual physical examination** (No)	1.26 (0.94, 1.69)	1.23 (0.89, 1.71)	1.84 (0.91, 3.72)	1.49 (0.69, 3.21)	0.96 (0.479, 1.88)	1.17 (0.56, 2.42)	0.61 (0.25, 1.46)	0.69 (0.23, 2.03)
**Cigarette smoking** (vs Nonsmoker)								
Former smoker	0.64^**^ (0.49, 0.84)	1.01 (0.69, 1.48)	0.57 (0.24, 1.36)	0.33 (0.09, 1.16)	1.72 (0.99, 2.99)	1.22 (0.49, 3.03)	0.49 (0.12, 1.91)	0.64 (0.07, 5.77)
Current smoker	0.40^***^ (0.32, 0.81)	0.70 (0.48, 1.01)	1.60 (0.70, 3.65)	1.69 (0.45, 6.30)	3.51^***^ (1.85, 6.67)	2.81 (1.00, 7.89)	0.73 (0.13, 4.18)	0.65 (0.06, 7.50)
**Alcohol intake** (Yes)	0.46^***^ (0.36, 0.58)	0.85 (0.63, 1.14)	0.86 (0.36, 2.02)	0.61 (0.20, 1.90)	1.75 (0.96, 3.19)	0.56 (0.24, 1.27)	0.79 (0.07, 8.90)	0.43 (0.02, 12.19)
**Daily tea** (Yes)	0.56^***^ (0.46, 0.67)	1.01 (0.79, 1.29)	0.82 (0.46, 1.47)	0.72 (0.34, 1.55)	2.05^***^ (1.35, 3.11)	1.08 (0.61, 1.92)	0.91 (0.33, 2.49)	0.94 (0.25, 3.55)
**Reading** (Yes)	0.44^***^ (0.34, 0.57)	0.84 (0.61, 1.15)	1.11 (0.43, 2.87)	1.18 (0.34, 4.16)	2.24^*^ (1.13, 4.46)	1.13 (0.48, 2.68)	3.25 (0.29, 36.81)	4.98 (0.24, 104.22)
**Play cards or mahjong** (Yes)	0.58^***^ (0.47, 0.72)	0.90 (0.71, 1.14)	0.63 (0.32, 1.26)	0.50 (0.22, 1.11)	1.32 (0.82, 2.12)	0.68 (0.38, 1.22)	0.38 (0.04, 3.53)	0.23 (0.02, 2.68)
**Physical exercise** (vs Almost never)								
Several times per week	0.75^*^ (0.58, 0.96)	0.73^*^ (0.55, 0.96)	1.67 (0.93, 2.99)	1.84 (0.94, 3.61)	1.30 (0.78, 2.14)	1.24 (0.70, 2.12)	2.34 (0.89, 6.13)	1.79 (0.55, 5.85)
Everyday	0.70^***^ (0.58, 0.85)	0.72^**^ (0.58, 0.90)	0.93 (0.54, 1.59)	1.04 (0.57, 1.89)	1.29 (0.84, 1.98)	1.01 (0.63, 1.63)	4.35^***^ (1.84, 10.29)	4.03^**^ (1.42, 11.46)
**Meeting with children** (vs Almost never)								
Several times per week	0.92 (0.74, 1.15)	0.94 (0.57, 1.16)	1.08 (0.60, 1.95)	1.13 (0.59, 2.14)	0.84 (0.53, 1.33)	0.71 (0.43, 1.17)	1.02 (0.33, 3.17)	1.56 (0.39, 6.22)
Everyday	1.04 (0.83, 1.31)	0.95 (0.73, 1.24)	1.06 (0.58, 1.95)	0.92 (0.45, 1.88)	0.70 (0.44, 1.13)	0.68 (0.39, 1.19)	1.38 (0.45, 4.24)	2.50 (0.56, 11.26)
**Neighbor interaction** (vs Almost never)								
Several times per week	0.70^*^ (0.51, 0.97)	0.81 (0.57, 1.16)	0.35^**^ (0.16, 0.76)	0.30^**^ (0.13, 0.74)	0.84 (0.44, 1.61)	0.75 (0.36, 1.54)	2.26 (0.64, 7.96)	2.34 (0.51, 10.81)
Everyday	0.59^***^ (0.46, 0.74)	0.67^**^ (0.51, 0.87)	0.56^*^ (0.32, 1.00)	0.52^*^ (0.27, 1.00)	1.07 (0.63, 1.83)	1.07 (0.59, 1.93)	3.00^*^ (1.10, 8.19)	2.85 (0.83, 9.82)
**Social participation** (vs Almost never)								
Several times per month	0.69^**^ (0.52, 0.90)	0.83 (0.62, 1.12)	1.39 (0.80, 2.41)	1.64 (0.85, 3.16)	0.93 (0.57, 1.52)	0.77 (0.44, 1.37)	1.21 (0.47, 3.14)	1.23 (0.34, 4.36)
Several times per week	0.75^*^ (0.58, 0.96)	0.90 (0.69, 1.19)	1.76 (0.82, 3.78)	2.56^*^ (1.06, 6.17)	2.62^**^ (1.44, 4.80)	2.14^*^ (1.08, 4.24)	0.81 (0.14, 4.62)	0.58 (0.07, 4.71)

^a^ Models for univariate analysis;

^b^ Models for multivariate analysis and adjustment for age and gender;

^***^p < 0.001;

^**^p < 0.01;

^*^P < 0.05

After all of the variables were entered into the model and additional adjustments for age and gender were applied, associations between frailty transitions and several factors weakened or disappeared. In particular, 1) those robust elders who had higher educational levels (compared with illiteracy, primary school: OR = 0.63, 95% CI: 0.51~0.78; junior high school or above: OR = 0.46, 95% CI: 0.33~0.64), still at work (OR = 0.60, 95% CI: 0.45~0.80), had more frequent physical exercise (compared with almost never, several times per week: OR = 0.73, 95% CI: 0.55~0.96; everyday: OR = 0.72, 95% CI: 0.58~0.90) and more neighbor interaction (compared with almost never, everyday: OR = 0.67, 95% CI: 0.51~0.87) predicted a lower risk of robust worsening, while having no shower facilities at home (OR = 1.39, 95% CI: 1.02~1.90) predicted a higher risk of robust worsening; 2) those prefrail elders who had more neighbor interaction (compared with almost never, several times per week: OR = 0.30, 95% CI: 0.13~0.74; everyday: OR = 0.52, 95% CI: 0.27~1.00) predicted a lower risk of prefrail worsening; 3) only more frequent social participation (compared with almost never, several times per week: OR = 2.14, 95% CI: 1.08~4.24) predicted a higher chance of prefrail improvement; and 4) among frail elders, only more physical exercise (compared with almost never, everyday: OR = 4.03, 95% CI: 1.42~11.46) predicted a higher chance of frailty improvement.

## Discussion

The present study showed that frailty status can deteriorate to a worse state (22.2%) but can also turn back to a better state (7.8%), though the majority stayed in the same state (70.0%). These results are similar to previous studies, [33, 38, 39] and continue to support the notion that frailty is a dynamic and reversible status in later life [40]. With increasing age, the risk of being frailty and frailty worsening were both higher. Older females have widely been found to have a higher prevalence of frailty, [2, 8, 41] which also showed in our study. In addition, the decline rate among robust and prefrail female elders was found to be higher than in males. However, after adjusting for lifestyle factors, it seemed that few differences in changes of frailty were consistent with previous studies [18, 39]. These findings suggest that females probably have worse health but a more protective lifestyle, which provides a reason for the health-survival paradox. A higher educational level, known to promote better psychological well-being and less dependence, [42] was found to be associated with decreasing frailty among baseline robust elders in a previous study [22] and in the current study.

This study found that, beyond stability, improvement in frailty status is possible, and those elders who were frail at baseline improved by 18.3% in males and 36.0% in females during a period of 2 years. Older adults who had physical exercise everyday had a four-fold chance of frailty improvement compared to those who almost never exercised, and those who had frequent physical exercise had a nearly 27% lower risk of robust worsening in this study. Various physical activities are associated with health, and a number of random control trials have demonstrated the benefit of exercise intervention for the frail elderly [30–32]. These encouraging findings support the idea that the maintenance of robustness and improvements in frailty status can be promoted through daily physical exercise.

Among the robust elders at baseline, those who worked and had more frequent neighbor interaction had a 40 and 33% decreased risk of frailty worsening, respectively. A comparison study found that older workers experience a greater sense of mastery than retired elders, [43] which may encourage additional correct choices related to health [44]. A sense of mastery, as a powerful psychological resource, is strongly linked to positive mental and physical health outcomes [44]. A lower frequency of social interactions is associated with feelings of loneliness and depression, [45] and neighbor interactions are also an important way for older adults to acquire social resources (e.g., health information, emotional and instrumental support), which may serve as important buffers for frailty worsening. This is consistent with the decreased frailty worsening by frequent neighbor interaction among prefrail elders. In addition, taking part in social activities frequently seemed to be beneficial for prefrail elders. The presence of health problems is more likely to attract their attention, and those who participated in social activities frequently may have more chances to access health knowledge, increase physical activity, and promote interpersonal relationships, all of which favor health [28].

Finally, we also found that baseline robust elders whose home does not have shower facilities predicted a higher risk of frailty worsening compared to those with showers. A lack of shower facilities at home could be an obstacle to personal hygiene, especially for older people, which may decrease their quality of life. However, it could simply reflect poor indoor conditions, which are likely to lead to adverse health outcomes.

### Strengths and limitations

Associations between lifestyle and frailty has been widely reported, but its influence on changes in frailty is unclear. This study is one of the few that has explored the relationship between various types of lifestyle factors and changes in frailty. This was a large longitudinal observational study with a very low dropout rate: 0.6% (*n* = 23), and to the best of our knowledge, this study is one of a few to demonstrate a relationship between lifestyle factors and changes in frailty.

However, several limitations should be also mentioned. First, all of the analytical data in the study were from self-reported measures, instead of more accurate laboratory and clinical tests. Even so, self-reported data have been suggested to be valid by numerous publications. Second, assessments for each lifestyle factor only referred to a single aspect, and many other aspects of these lifestyle factors have not been considered. For instance, 1) other facilities or characteristics related to assisted ageing could be considered in the assessment of indoor conditions for the elderly; 2) other dietary habits (e.g., vegetable intake, fruit intake) could also be included; and 3) for social participation (physical exercise, interactions, and activities), the frequency of participation is important, but the satisfaction and style of participation should not be ignored [46, 47]; these are potential reasons for the different results among different frailty statuses. Finally, the study sample was drawn from the Shanghai area, which is one of the most developed cities in China, and it might not represent other parts of China.

## Conclusions

This longitudinal population-based study suggests that the status of frailty was reversible among community-dwelling elderly, and lifestyle protective factors were likely to decrease the risk of frailty worsening. Although several health behaviors were not separately significantly related to changes in frailty, cumulative protective factors are associated with a lower risk of frailty deterioration or mortality, as well as a greater chance of recovery [33]. Of the lifestyle predictors, many more factors were found to be associated with changes in frailty among robust and prefrail elders than frail elders, which suggests that early identification and intervention have a bigger chance of maintaining or delaying declines related to frailty status. These findings help health practitioners to recognize susceptible individuals in a community and provide health promotional planning to target aged populations.

## Supplementary information

**Additional file 1:.** Components of the frailty index (FI) in the study

## Data Availability

The data that support the findings of this study are available from The National Center for Chronic and Noncommunicable Disease Control and Prevention but restrictions apply to the availability of these data, which were used under license for the current study, and so are not publicly available. Data are however available from the authors upon reasonable request and with permission of The National Center for Chronic and Noncommunicable Disease Control and Prevention.

## Abbreviations

CI: Confidence interval
FI: Frailty index
FP: Frailty phenotype
OR: Odds ratio
SD: Standard deviation
SPSS: Statistical Package for the Social Sciences
USA: United States of America
