# Supplementary material for: Changes in frailty among community-dwelling Chinese older adults and its predictors: evidence from a two-year longitudinal study
Source: BMC Geriatr. 2020 Apr 10;20:130. doi: 10.1186/s12877-020-01530-x (PMC7146912; doi:10.1186/s12877-020-01530-x)
Supplement: Supplementary file 1 — Additional file 1:. Components of the frailty index (FI) in the study [file 12877_2020_1530_MOESM1_ESM.docx]

| **Additional file 1**: Components of the frailty index (FI) in the study | | | | | | | | |
| --- | --- | --- | --- | --- | --- | --- | --- | --- |
| Item | Baseline (2015) | | |  | 2-year follow up (2017) | | | Decision ^b^ |
|  | r ^a^ | n | % |  | r ^a^ | n | % |  |
| Presence of current diseases |  |  |  |  |  |  |  |  |
| •  Hypertension | .062^**^ | 1764 | 44.23 |  | .063^**^ | 1842 | 47.99 | O |
| •  Diabetes mellitus | -.023 | 400 | 10.03 |  | -.017 | 443 | 11.54 | × |
| •  Coronary heart disease | .090^**^ | 274 | 6.87 |  | .096^**^ | 292 | 7.61 | O |
| •  Stroke | .088^**^ | 237 | 5.94 |  | .112^**^ | 420 | 10.94 | O |
| •  Lung disease | .086^**^ | 223 | 5.59 |  | .076^**^ | 248 | 6.46 | O |
| •  Asthma | .013 | 56 | 1.40 |  | .011 | 71 | 1.85 | × |
| •  Kidney disease | .000 | 57 | 1.43 |  | .003 | 56 | 1.46 | × |
| •  Tuberculosis | .026 | 48 | 1.20 |  | .011 | 30 | 0.78 | × |
| •  Glaucoma | .027 | 32 | 0.80 |  | .015 | 32 | 0.83 | × |
| •  Cataract | .202^**^ | 739 | 18.53 |  | .156^**^ | 897 | 23.37 | O |
| •  Arthritis | .010 | 459 | 11.51 |  | .005 | 358 | 9.33 | × |
| •  Cervical spondylosis | -.053^**^ | 280 | 7.02 |  | -.037^*^ | 214 | 5.58 | × |
| •  Herniated disk | -.048^**^ | 257 | 6.44 |  | -.047^**^ | 269 | 7.01 | × |
| •  Cancer/malignant tumor | .015 | 87 | 2.18 |  | -.004 | 123 | 3.20 | × |
| Ability in the activities of daily living |  |  |  |  |  |  |  |  |
| •  Difficult in feeding | .052^**^ | 25 | 0.63 |  | .087^**^ | 64 | 1.67 | O |
| •  Difficult in dressing | .068^**^ | 39 | 0.98 |  | .112^**^ | 89 | 2.32 | O |
| •  Difficult in bathing | .063^**^ | 42 | 1.05 |  | .137^**^ | 133 | 3.47 | O |
| •  Difficult in walking | .046^**^ | 24 | 0.60 |  | .084^**^ | 66 | 1.72 | O |
| •  Difficult in toileting | .053^**^ | 25 | 0.63 |  | .100^**^ | 110 | 2.87 | O |
| •  Difficult in transferring | .037^*^ | 19 | 0.48 |  | .100^**^ | 88 | 2.29 | O |
| •  Difficult in continence | .038^*^ | 13 | 0.33 |  | .059^**^ | 35 | 0.91 | O |
| •  Difficult in telephone use | .299^**^ | 334 | 8.38 |  | .307^**^ | 390 | 10.16 | O |
| •  Difficult in shopping | .174^**^ | 124 | 3.11 |  | .236^**^ | 254 | 6.62 | O |
| •  Difficult in cooking | .144^**^ | 92 | 2.31 |  | .189^**^ | 188 | 4.90 | O |
| •  Difficult in housekeeping | .155^**^ | 104 | 2.61 |  | .194^**^ | 193 | 5.03 | O |
| •  Difficult in doing laundry | .141^**^ | 96 | 2.41 |  | .196^**^ | 193 | 5.03 | O |
| •  Difficult in using transportation | .232^**^ | 201 | 5.04 |  | .303^**^ | 412 | 10.73 | O |
| •  Difficult in handling medications | .095^**^ | 43 | 1.08 |  | .098^**^ | 85 | 2.21 | O |
| •  Difficult in handling finances | .134^**^ | 105 | 2.63 |  | .176^**^ | 163 | 4.25 | O |
| Physical and neurological signs |  |  |  |  |  |  |  |  |
| •  Urinary incontinence | .045^**^ | 16 | 0.40 |  | .073^**^ | 24 | 0.63 | O |
| •  Impaired hearing | .080^**^ | 137 | 3.44 |  | .072^**^ | 138 | 3.60 | O |
| •  Irregular gait pattern | .143^**^ | 157 | 3.94 |  | .188^**^ | 335 | 8.73 | O |
| •  Difficult in buttoning up | .039^*^ | 24 | 0.60 |  | .038^*^ | 48 | 1.25 | O |
| •  Writing change | .265^**^ | 1614 | 40.47 |  | .263^**^ | 1700 | 44.29 | O |
| •  Voice weaken | .083^**^ | 123 | 3.08 |  | .133^**^ | 337 | 8.78 | O |
| •  Facial change | .070^**^ | 49 | 1.23 |  | .074^**^ | 220 | 5.73 | O |
| Cognitive and mental symptoms |  |  |  |  |  |  |  |  |
| •  Memory worse than last year | .104^**^ | 1890 | 47.39 |  | .106^**^ | 1888 | 49.19 | O |
| •  Date orientation impairment | .181^**^ | 297 | 7.45 |  | .204^**^ | 354 | 9.22 | O |
| •  Time orientation impairment | .092^**^ | 190 | 4.76 |  | .128^**^ | 290 | 7.56 | O |
| •  Trouble remembering things | .101^**^ | 216 | 5.42 |  | .170^**^ | 396 | 10.32 | O |
| •  Dyscalculia | .144^**^ | 220 | 5.52 |  | .168^**^ | 406 | 10.58 | O |
| •  Often repeating | .051^**^ | 99 | 2.48 |  | .093^**^ | 207 | 5.39 | O |
| •  Difficult in operating remote control | .150^**^ | 103 | 2.58 |  | .169^**^ | 175 | 4.56 | O |
| •  Impaired judgement | .034^*^ | 96 | 2.41 |  | .042^**^ | 142 | 3.70 | O |
| •  Loss of interest | .045^**^ | 76 | 1.91 |  | .063^**^ | 212 | 5.52 | O |
| ^a^ The spearman correlation coefficients between items with age;  ^b^ The circle presents eligible items and the cross presents ineligible items;  ^**^ p<.01;  ^*^ p<.05. | | | | | | | | |
